# Barriers to providing internet-based home care services for urban older adults in China: a qualitative study of the service providers

**DOI:** 10.1186/s12877-023-04028-4

**Published:** 2023-05-23

**Authors:** Caiyun Qi, Yuan Wang, Xiaonan Qi, Yunhe Jiao, Chuanqi Que, Yufei Chen

**Affiliations:** 1 Department of social work, Shandong University, Jinan, China; 2 Department of labor and social security, Jilin University, Qianjin Street 2699, Changchun, China; 3 Department of management, School of applied technology and health industries, Anshan Normal University, Anshan, China; 4 department of social work, The Chinese University of Hong Kong, Hong Kong, China

**Keywords:** Internet-based Home Care Services, Service providers, Urban older adults, Barriers, Qualitative study

## Abstract

**Background:**

Due to the increasingly aging population in China and the changes in social and family structure, older adults’ care problems are becoming more and more prominent. To meet the home care needs of urban older adults, the Chinese government has launched Internet-Based Home Care Services (IBHCS). Although this model innovation can significantly relieve care problems, more and more evidence shows that there are many barriers in the process of IBHCS supply. The current literature is mostly from the perspective of the service users, and there are very few studies on the experience of service providers.

**Methods:**

In this study, we took a qualitative phenomenological approach and used semi-structured interviews to investigate service providers’ daily experiences and the barriers they encounter. A total of 34 staff from 14 Home Care Service Centers (HCSCs) were included. Interviews were transcribed and analyzed using thematic analysis.

**Results:**

We identified the barriers that service providers encounter in IBHCS supply: (1) bureaucratic repression: unreasonable policy plans, harsh assessment, excessive paperwork, different preferences of government leaders, and obstacles caused by COVID-19 control lead to a shift of focus in their work; (2) profitability crisis in the market: high service costs, dampened effective demand, government intervention in setting prices, and parent companies’ excessively high sales targets hinder the service supply process; (3) client-related challenges: the crisis of confidence, the dilemma of popularizing new technology, and communication barriers lead to rejection by older adults; (4) job dissatisfaction: low and unstable salary, heavy tasks, poor social acceptance of occupations, and lack of professional value reduce work enthusiasm.

**Conclusion:**

We have investigated the barriers faced by service providers when providing IBHCS for urban older adults in China, providing empirical evidence in the Chinese context for the relevant literature. In order to provide IBHCS better, it is necessary to improve the institutional environment and market environment, strengthen publicity and communication, target customer needs, and adjust the working conditions of front-line workers.

**Supplementary Information:**

The online version contains supplementary material available at 10.1186/s12877-023-04028-4.

## Background

The increasingly aging population and the large proportion of older adults pose great challenges to the whole world. In 2019, 703 million people worldwide were aged 65 or older, representing approximately 9% of the global population [1]. China is one of the countries with the largest elderly population and the fastest aging rate [2]. According to the seventh national census in 2020, China’s population aged 65 and above was 190 million, accounting for 13.5% of the total population [3], higher than the world average. The problem of rapid aging has attracted widespread attention to older adults’ health status and care needs [4]. In addition, social changes in China, such as urbanization, reduced family size, and increased female employment rate, further lead to more major problems in the care of older adults [5, 6]. Therefore, how to meet the needs of older adults for care services has become an important issue facing the world, especially in China.

Worldwide, providing home care services is essential to urban older people. Available research evidence suggests that older adults generally prefer to receive care at home [7, 8]. In the United States, Canada, and other western countries, home care has become an effective way to deal with the aging problem [9, 10], and among which, since the 1980s, with the development of emerging information technologies such as the Internet, cloud computing, and intelligent hardware, smart home for the elderly has become one of the most important ways. In different countries and regions, it is also called e-Home care [11], Ambient Assisted Living (AAL) [12, 13], Telehomecare [14], etc. Smart home for the elderly aims to provide remote management and various assistance for older adults in need [15]. It achieves its service objectives in two ways. First, it is outfitted with unobtrusive and non-invasive environmental and physiological sensors and actuators to remotely manage and monitor the home environment and vital physiological signs and activities of older adults. The second is to help older adults communicate with healthcare facilities and caregivers through intelligent equipment to respond to their needs in time [16]. Smart home for the elderly creates an intelligent care environment for older adults with fewer human resources and lower costs [17]. Systematic literature review shows that it has four specific benefits for users: health-related benefits, environmental benefits, financial benefits, and psychological welling and social inclusion [18].

Although the practice in different areas is various [15], the existing literature has shown that the successful acceptance of smart home for the elderly is still a common challenge. Why is the development of smart home for the elderly not satisfactory? From the service users’ perspective, first, an inadequate understanding of user needs is seen as the main factor [19]. The reason is that it is often dominated by suppliers providing a technology-push, rather than a demand-pull approach [20]. Second, the availability barriers in smart home for the elderly also lead to user disappointment. The need for more product reliability and technical complexity often makes older adults refuse to use it [21, 22]. The third is the ethical issue. Privacy (information privacy and physical privacy), autonomy (independence, informed consent, and user-centered control), safety guarantee, fairness, and concerns about reduced human contact have been widely discussed [23–26]. In particular, the lack of relevant legislation has exacerbated users’ distrust [27]. The fourth is financial difficulties. The technology’s price, installation and maintenance costs directly lead to older adults’ rejection [18, 28]. Fifth, older adults are more committed to established habits and strongly resist new technologies that change their behavior and lifestyle [18, 29]. From the suppliers’ approach, the diversification of supply entities makes the poor compatibility of different devices and also increases the difficulty of promoting smart home for the elderly [30]. In addition, care managers’ doubts about products and functions, knowledge and skills gap, and the complexity of clients make their work more difficult [11].

The smart home for the elderly in China began late and was first included in the policy agenda in 2008. It is often called Internet-based Home Care Services (IBHCS) and spreads in urban areas. In 2015, implementing the “Internet plus” plan provided overall guidance for the development of IBHCS. In 2017, the *Action Plan for The Development of Smart Health and Elderly Care Service Industry (2017–2020)*, released by the Ministry of Industry and Information Technology and the Ministry of Civil Affairs, put forward a specific action plan on how to apply IBHCS to older adults. At present, the service content and process of IBHCS are shown below. First of all, the government undertakes the design and implementation of IBHCS in various regions, including establishing the “virtual nursing home” (an information service platform) to support care enterprises with technological infrastructure to join and merge into resident communities, determining the object and scope, deciding the specific production mode, defining the quantity and quality standards of service supply, and supervising service providers. Secondly, older adults can indirectly express their needs through wearable medical devices or directly through online applications. Thirdly, the “virtual nursing home” includes the information of older adults, such as individual health information and service needs information, into the virtual service system. Through the analysis of information, the supplier and demander are reasonably coordinated. Finally, the service provider exports corresponding care services to older adults. As the most essential service provider, elderly care enterprises establish a Home Care Services Center (HCSC) in every community to provide related services, including life care services (laundry, food delivery, water, clothing, haircut, shopping, toilet, floor mopping, information consultation, etc.), medical care services (sending medicine, taking medicine, physical indicators, health lectures, health training, rehabilitation guidance, etc.), spiritual comfort services (chat, psychological counseling, walking, online chat, online dating, etc.), emergency assistance services (emergency care, automatic alarm, remote monitoring, etc.) [31–34]. Please note that these services may be free or charged. On the one hand, through government purchase services and financial subsidies, HCSC provides free health lectures, health training, social activities and other services for all older adults, or provides a certain amount of “home care service voucher” or “cash nursing benefits” for some eligible poor, severely disabled, dementia older adults and older people who lost their only child or living alone. On the other hand, after exceeding the free quota of vouchers or cash benefits, HCSC allows older adults to purchase the service items in the government purchase contract at a lower price than the market price. In addition, HCSC also provides fully market-oriented charging services. The above details vary slightly from region to region.

Compared with other countries, it can be seen that China’s IBHCS has noticeable differences. First, displaying “intelligence,“ such as remote management of household automation equipment, is not enough, but the simple use of Internet technology [35]. Second, the IBHCS under authoritarian China is mainly driven by top-down policies rather than technology-driven or bottom-up demand-driven [36]. Therefore, the service providers are obviously influenced by the government’s value orientation and bureaucratic task, which determines the service area, service object, and service depth to a large extent [37]. In this regard, some scholars pay attention to the obstacles existing in IBHCS in the Chinese context. Similar to foreign research results, the identified barriers can be classified as: poor user-friendliness of Internet-related devices [38], low adaptation to new things [39], single service type, poor service quality [40], and concerns about data security issues such as privacy [41, 42]. Meanwhile, a few Chinese types of research also focus on the institutional environment of service providers’ service supply. Qu found that high dependence on government support led to the alienation of work objectives [43]. Chen & Shao proposed that authoritative management led to the continuous infiltration of administrative power into the market organization, resulting in the blurring and even confusion of the boundary between the market and the government [44]. In general, compared with studies in western countries, this issue is less discussed in the Chinese context. Moreover, these studies are more based on the demand side. Although they reflect the current situation that the needs of older adults cannot be met, it is difficult to provide a more precise and deeper response to why the service providers cannot meet these needs and how they should solve this dilemma in the Chinese context. Meanwhile, most existing studies are just a simple summary of the relevant barriers, lacking sufficient empirical evidence.

The purpose of this study is to explore the experience of service providers in implementation of IBHCS in the context of China. Through qualitative research methods, we demonstrate a comprehensive dimension of the barriers to IBHCS for urban older adults. This study can supplement the knowledge of the service provider perspective in the study of IBHCS practice barriers, and provide a unique Chinese case for the related research of smart home for the elderly.

## Methods

### Design

This study was designed using a qualitative phenomenological approach [45]. This approach provides a way to study complicated phenomena in human society, focusing on human experience and reflecting on the meaning behind it [46]. In our study, it can help us gain a deeper understanding of the barriers in the complex phenomenon of providing IBHCS to urban seniors in China. To present more information, we conducted interviews with 34 staff members from 14 HCSCs in Changchun, China. Interviews were transcribed and analyzed using thematic analysis. The Consolidated Criteria for Reporting Qualitative Study (COREQ) checklist was followed (see Additional file 1) [47].

### Sampling and recruitment

The study was conducted in Changchun, which is located in northeast China. The city’s residents aged 65 and above account for 14.15% of its total population, making it one of China’s top 10 aging cities. With the intention of solving the aging problem, the city became one of the earliest pilot cities to launch IBHCS. We recruited participants through the HCSCs; the HCSCs worker list was obtained through the largest senior care enterprise in Changchun. We selected potential participants from the list and invited them by phone or email. We provided general information related to the study, including the purpose, process, duration, and research schedule, and made it clear that participation was voluntary. After informed consent was obtained, the interviews were arranged.

Participants were selected by purposive sampling [48]. The researchers used the following criteria: (1) participants are currently employed by an HCSC in the community; and (2) participants have at least one year of working experience involved in management or service delivery. To construct a comprehensive understanding, we used the maximum variation strategy to balance the sample according to staff characteristics [48, 49], such as age, gender, educational attainment, job position, and years in work. Sampling, data collection and data analysis ran concurrently [50]. After 32 interviews, no new themes emerged, suggesting that the data had reached saturation [51, 52]. We conducted two more interviews, without finding new information, after which we stopped recruiting. In the end, 36 participants from 14 HCSCs (distributed in six major urban areas of Changchun: Kuancheng District, Erdao District, Nanguan District, Lvyuan District, Chaoyang District, and Shuangyang District) accepted the invitation and participated in the study; two of these participants withdrew because of problems in finding a suitable time for interviews.

### Data collection

A total of 34 face-to-face interviews were conducted, with an average duration of 38 min (between 19 and 90 min). Interviews give access to interviewees’ stories that are complex expressions of personal experiences and their reflections [53]. To ensure that the interviews were administered consistently, the interviewees were interviewed by the same researcher (XNQ) from May to September 2021. XNQ is a master student in the field of elderly policy, and is very familiar with IBHCS; she is also well trained in qualitative data collection. Other authors provided necessary guidance and supervision. To encourage participants to express themselves freely, all interviews were conducted in an independent office at HCSCs without the presence of third parties. Prior to the interviews, the researcher reiterated the general information about the study to the participants, asking for permission for the interviews to be recorded. Meanwhile, participants were informed that they could stop their interview and quit the study at any time. During the interview, the researcher listened carefully and observed and recorded the interviewees’ gestures, facial expressions, and other non-verbal signs. After the interviews, the researcher kept reflective logs.

To facilitate deeper dialogue, we constructed a semi-structured interview guide by the five-step method of Kallio, Pietelä, Johnson & Kangasniemi [54]: (1) identifying the prerequisites for using semi-structured interviews; (2) retrieving and using previous knowledge; (3) formulating the preliminary semi-structured interview guide; (4) pilot testing interview guide; and (5) presenting the complete semi-structured interview guide. The interview guide was drafted based on our research questions and literature review (including the service delivery process [31–33], interview guide [55–57], and research results [36, 43, 44, 58–61] of relevant researches), and was discussed in depth by the research team. The effectiveness of the designed questions is achieved by answering the following five questions: (1) Why do you ask this question? (2) What is the question? (3) How do you keep your interview questions open rather than pointed? (4) How did you use the interviewees’ own “host culture” to form a guide? (5) Are the way of expression and sequence of the questions appropriate? [62]. To ensure it was easily understandable, the interview guide was tested with two pilot interviews, and the revised version was used in the formal interviews. The interview guide consisted of open questions focused on subjective experiences, including participants’ work experiences, interactive experiences with other stakeholders, and perceptions of barriers to service delivery. The interview guide is reproduced in the Additional file 2. In addition to this guide, the researcher also prompted participants to “Tell me more” and “Can you explain that further?“ to obtain additional information.

### Data analysis

All interviews were transcribed word-for-word into Chinese through iFlytek Listening (voice-to-text software) and then analyzed. The themes and sub-themes identified in the analysis, and supporting quotations, were translated into English to be included in the presentation of the research results. The fourth author (YHJ) repeatedly listened to the recordings to further improve the transcripts; the first author (CYQ) reviewed the accuracy of the transcriptions and processed them anonymously.

In this study we used the inductive thematic analysis method to analyze the data [63, 64]. Work proceeded according to the six steps described by Braun and Clarke [64]: (1) familiarizing ourselves with the data, (2) generating initial codes, (3) searching for themes, (4) reviewing identified themes, (5) defining and naming themes, and (6) producing the reports. All authors repeatedly read and reflected on these transcripts in this process, independently identifying and condensing the meaning units in the text concerning the barriers. Then, the entire research team met to compare and discuss any differences until consensus was reached on how to classify codes. The relationship between the different codes was then analyzed to form the themes. This process was iterated in parallel with sampling and data collection, and emerging themes were repeatedly overlapped and merged until no new themes could be identified [64]. After discussion and repeated review by team members, a total of four themes and 16 sub-themes were generated from the data.

### Rigor

To ensure the trustworthiness of the study, we used the four criteria specified by Lincoln and Guba [65]. First, to improve the credibility of the research, researchers underwent strict training, and reflective logs and interview records were completed following standardized procedures. Second, transferability was ensured through an in-depth and detailed description of the research process and participants. Third, to achieve dependability, we collected as much raw material as possible and reviewed the transcripts several times; these were coded independently by each investigator, and we discussed the transcripts extensively. Finally, confirmability was reached by using rich quotations to illustrate the themes and sub-themes from the interviews (see Additional file 3).

### Ethical considerations

The entire study followed Institutional Review Board (IRB) procedures and considered the strict requirements of confidentiality, non-harm, and informed consent for participants. We obtained ethical approval from the Academic Committee of the School of Philosophy and Sociology of Jilin University.

## Results

The sociodemographic characteristics of 34 participants are shown in Table 1. Their job titles are: manager (3, 8.8%), center manager (9, 26.5%), center staff (12, 35.3%), domestic worker (5, 14.7%), and nursing worker (5, 14.7%). Most of the participants were between 30 and 39 years old (14, 41.3%), followed by 20 to 29 years old (8, 23.5%), and were mainly female (24, 70.6%). The education level of more than half of the participants was undergraduate (20, 58.8%). The average years in employment were 3.9, with nearly 80% of the participants having worked for less than six years.

**Table 1:** Characteristics of participants(n = 34)

Characteristics	N (%)
Age, year	
20–29	8 (23.5)
30–39	14 (41.3)
40–49	6 (17.6)
50–59	6 (17.6)
**Gender**	
Female	24 (70.6)
Male	10 (29.4)
**Educational attainment**	
Middle school	1 (2.9)
High school	2 (5.9)
College	6 (17.6)
Bachelor	20 (58.8)
Master	5 (14.8)
**Job title**	
Manager	3 (8.8)
Center manager	9 (26.5)
Center staff	12 (35.3)
Domestic worker	5 (14.7)
Nursing worker	5 (14.7)
**Years in employment**	
1	3 (8.8)
2	6 (17.6)
3	8 (23.5)
4	4 (11.8)
5	5 (14.7)
6	4 (11.8)
7	3 (8.8)
8	1 (2.9)

Among the barriers to service providers when offering IBHCS, we identified the following four themes: bureaucratic repression, profitability crisis in the market, uncooperative clients, and career dissatisfaction. See Table 2 for details.

**Table 2:** Themes and sub-themes

Themes	Sub-Themes
Bureaucratic repression	Unreasonable policy plans
	Harsh assessment
	Excessive paperwork
	Different preferences of government leaders
	Obstacles caused by COVID-19 control
Profitability crisis in the market	High service cost
	Dampened effective demand
	Government intervention in setting price
	Parent companies’ excessively high sales targets
Client-related challenges	Crisis of confidence
	Dilemma of popularizing new technology
	Communication barriers
Career dissatisfaction	Low and unstable wages
	Heavy tasks
	Poor social acceptance of occupations
	Lack of professional value

### Bureaucratic repression

Service providers who are included in the government’s purchasing system need to act under government regulations. They believe that bureaucratic work processes, such as what they deem to be unreasonable policy plans, harsh assessments, lots of paperwork, different preferences of government leaders, and obstacles caused by COVID-19 control (Table 2) hinder service delivery.

#### Unreasonable policy plans

To realize the promotion of IBHCS, the government has designed a series of policy plans, some parts of which have caused great difficulties in its implementation. Interviewees pointed out that in the detailed implementation rules of IBHCS, some of the service contents, provision methods, and equipment matching are uniformly stipulated. However, uniform requirements are not suitable for the specific situation of every HCSC, resulting in a large number of wasted resources. For example, the contract for the government purchase services stipulates that every HCSC must be equipped with cleaning and nursing beds, massage machines, and so on. But these devices are only put aside and barely used by older adults.

*This room is equipped with cleaning and nursing beds, massage machines, etc. This equipment is necessary for participating in government purchase service projects. However, no one uses it. This room are used as a warehouse. (Participant 9, center staff)*

*These (Internet) types of equipment are not used in the service but mainly used to finish administrative work. They are invested in qualifying for government purchasing public services. However, most of them are not very useful, so they are just placed here and left unused. (Participant 3, center staff)*

At the same time, participants also felt that uniform requirements limited their ability to adapt services to care needs in a timely manner. One center manager described her experience:

*Now the service is not very targeted. For example, there are a lot of mental consolation needs in our community. However, the government requires us to meet these needs mainly by holding regular lectures, which is not very effective… We cannot change it… (Participant 8, center manager)*.

#### Harsh assessment

Rigorous assessment and penalties constrain service providers’ energy to provide care services. The government provided financial support for HCSCs in the qualified suppliers’ list and at the same time formulated strict assessment rules, including six first-level indicators (human resources, infrastructure, environment, intelligent platforms, risk prevention, and service satisfaction) and dozens of specific indicators. If the indicators are not fulfilled, service providers face criticism, rectification, or even cancellation of qualification. Participants expressed great concern about how punishment could be avoided without affecting the provision of services. In the excerpts, the respondents reported actions that prioritize completion of assessments over the satisfaction of the needs of the elderly; here is an example:

*The meal services, we have stopped now. Because the assessment requires safe, hot meals, many older adults live far away, and it’s challenging to accomplish… It’s too risky, and we could even lose the qualification (government procurement of services). (Participant 15, manager)*

#### Excessive paperwork

Service providers are subject to bureaucratic work processes in which much paperwork not only occupies their service time, but also leads to a scramble for figures. Because work performance is presented primarily through paperwork, filling in a large number of forms, repetitive arrangement of documents, media reports and propaganda, and recording of video and pictures have become the main components of service providers’ work. Furthermore, their performance is mainly evaluated by figures, such as the number of older adults served and the number of times served, which leads them to focus more on quantity rather than quality. Therefore, service providers were busy presenting good numbers and their evidence, making it difficult for some interviewees to accept. As many center managers and center staff have mentioned, the needs of elderly people and the quality of services they receive seem to be ignored:

*Now there are too many checks, and there is no time for service. Different departments come to us to fill in forms…the same contents will be filled in several times… Whether we do it or not, we must have all the materials needed to look good. (Participant 6, center manager)*

#### Different preferences of government leaders

Different government leaders have different preferences. As a result, some sub-projects in IBHCS are always unsustainable, which affects the effect of service supply. From the research, we found that many older adult customers were not selected through market competition, but appointed by government departments. The number of older adults in different communities is different, and the profits are also very different. In China, the Civil Affairs Bureau refers to a functional department in charge of social and administrative affairs. The Sub-District Office is the administrative body of “Jiedao” (the lowest administrative level in China). The Community Neighborhood Committee is the resident autonomous organization, but it belongs to the jurisdiction of the Sub-District Office and has a strong administrative mandate. These three departments/organizations are the main ones involved in the IBHCS project. In order to win over elderly communities, three managers all admit that they need to establish various formal and informal relationships with the leaders of the Civil Affairs Bureau, the Sub-District Offices and the Community Neighborhood Committees. One of the main ways is to follow these leaders’ preferences to gain their favor. However, leaders’ different preferences create barriers to the sustainability of IBHCS, as this excerpt from one manager shows:

*They (the community neighborhood committee) change leaders all the time, almost every two years. A new broom sweeps clean. The new leadership will naturally consider whether the existing project is practical, or good, whether it is necessary… We have to listen to their ideas to stop some projects and develop some new ones. (Participant 20, manager)*

#### Obstacles caused by COVID-19 control

The COVID-19 pandemic has hit IBHCS hard. China has implemented very strict prevention and control measures for COVID-19, which require reducing everyone’s movement. Many interviewees felt that the Community Neighborhood Committee thought that providing care services for the elderly was considered secondary to the more critical COVID-19 prevention. As a result, in order to avoid the governance pressure caused by the epidemic outbreak in the community, they have banned many offline services of IBHCS, which needs both “online” and “offline” coordination; meanwhile, the COVID-19 pandemic has disrupted the “offline” service chain, leading to the inevitable loss of the customer base:

*Due to the COVID-19 pandemic, the policy stipulates that door-to-door services for the elderly will be suspended. Older adults don’t have a health code, so all these community activities such as free clinics have been suspended… so much work cannot be carried out now, and customer stickiness goes undiagnosed. Gosh, it is too hard… (Participant 11, center manager)*.

In addition, the ban on door-to-door services has made it difficult for domestic workers and nursing workers to get a salary, which has also triggered their departure. The staff exodus makes the already tricky service delivery team situation even worse:

*Many people left their jobs during the COVID-19 pandemic. Once they leave, we need new employees to replace them and need to train the new group… It’s too difficult. Several HCSCs have closed down. (Participant 10, manager)*

### Profitability crisis in the market

IBHCS is also inevitably affected by the care market environment. Factors such as high service cost, dampened effective demand, government intervention in setting prices, and parent companies’ excessively high sales targets, limit the sustainable development of HCSCs (Table 2).

#### High service cost

As the Internet and related intelligent equipment are involved in home care, the cost of these services has increased greatly. Participants mostly believe that the application of the Internet is not ideal, and that high consumption costs have seriously hindered market expansion. So they have no choice but to reduce some intelligent elements in the service to reduce costs:

*At first, we all provided smart wristbands and smart sensors, but now we don’t, because it’s a money-losing project… Developing a wristband requires too much money. A wristband costs more than 300 Yuan, and the older people think it is too expensive, so no one buys it. (Participant 5, center staff)*

#### Dampened effective demand

Influenced by traditional culture and traditional consumption concepts, China still focuses on free family care and state pensions, leading to the unpopularity and low effective demand for selling market services. Respondents describe their frustration in promoting IBHCS, particularly the sharp contrast between the great enthusiasm of older adults for free services and their apparent rejection of charged-for services. An excerpt from a center staff member illustrates this:

*In the beginning, health-monitoring equipment for medical care services was free, and everyone was willing to install it. When they are told they need to pay 160 yuan for installation, many older people are not willing to do it. Some said it’s not helpful, some worried about privacy… There are all sorts of reasons. Actually, they just don’t want to spend money. (Participant 9, center staff)*

Although HCSCs offer some free care services, they are not available to all seniors. One of the most critical free IBHCS sub-projects is the Home Care Service Voucher. The government provides a fixed amount of service vouchers for authorized older adults, who can consume them at designated HCSCs (the consumption is free for older adults because the government pays it). However, the eligibility criteria for Home Care Service Vouchers are very narrow, and the subsidies are also low. The interviewees described how the care demand from this project was limited:

*There are a lot of older adults who need care services. It seems there is much demand. However, they do not meet the eligibility criteria for a free Home Care Service Voucher, and the elderly have low pensions, so few of them are willing to buy it. (Participant 14, center manager)*

#### Government intervention in setting price

In order to cultivate the elderly care market, the government has adopted the purchase of public services. However, the low agreement price in the purchase contract hindered HCSCs and their affiliated enterprises from providing services. The research showed that the vast majority of HCSCs’ transactions come from the government purchase service, especially the aforementioned Home Care Service Voucher program. One interviewee said:

*When the government bids, HCSCs must offer care services according to the bidding price. Some prices are set by enterprises and more often by the government. The government has greater bargaining power. (Participant 11, center manager)*

There are two kinds of prices for the same service in the service price list: market price and government purchase price, the latter being lower than the former, significantly reducing profit margins. Therefore, HCSCs must cancel some services that are less profitable or even losing money. The following excerpt describes the disconnection between service providers providing services on demand and the businesses’ priority of profitability:

*The government price is too low for us to make money. For those with relatively low demand, with only 10–20 orders a year, we cannot reduce labor costs. (Participant 24, center manager)*

#### Parent companies’ excessively high sales targets

Challenging sales targets have led to goal displacement and made timely delivery of care services hard to accomplish. These HCSCs belong to several large care enterprises. According to the interviewees’ descriptions, these enterprises set monthly sales targets and rank their HCSC employees to motivate them. However, judging from the work experience of respondents, the targets are too high to achieve, although they did say they understood the enterprises’ behavior because *“the enterprise needs to make money.“* But they felt they were under tremendous pressure. Because of this barrier, HCSC staff changed the goals of IBHCS to the detriment of older adults’ rights. Among them, the purchasing agent service in the life care service is the most mentioned. IBHCS encourages the establishment of community supermarkets for older adults, especially those with difficulty walking. Older adults can easily buy cheaper vegetables, fruits, and other daily necessities online (door-to-door delivery) or offline. But in fact, the majority of buyers in many community supermarkets are young people. In the following excerpt, a center staff member describes the process of this goal replacement:

*At first, the purchasing agent service was aimed at older adults, but they do not have strong purchasing power like young people do. We were finding it hard to finish the task every month. So we opened it up to young people for them to buy. Since then, the goal is easily accomplished. (Participant 32, center staff)*

### Clients-related challenges

Although IBHCS can greatly improve living conditions for older adults, service providers still suffer from client non-cooperation, including the crisis of confidence, the dilemma of popularizing new technology, and communication barriers (Table 2).

#### Crisis of confidence

The difficulty of IBHCS supply lies in establishing customer trust. Many participants found that, on the one hand, older adults’ traditional attitudes lead them to attach importance to privacy and resist things like Internet applications. On the other hand, negative media opinion has aggravated clients’ doubts about commercial elderly care services. A crisis of confidence makes it hard for service providers to do their job:

*We are involved as a third-party organization. People don’t recognize us, thinking we are liars. When we ask to use older adults’ mobile phones to register and fill in their basic information, they refuse to comply. (Participant 26, center manager)*

*Media opinion has a strong impact. There is much negative news about abuse of the elderly, or related subjects. In fact, such abuse is really rare. But the Chinese people love to pay attention to negative news involving accidents or something terrible. This makes some older people and their children distrust us greatly. (Participant 26, center manager)*

#### Dilemma of popularizing new technology

Due to years of life concepts and habits, older adults tend to adopt the traditional care model. They find it difficult to adapt to IBHCS, the emerging care model, which hinders service delivery. Almost all respondents described the same feeling that their clients had less access to the Internet and were less able to learn how to use new technology:

*The willingness of older adults to accept Internet products is not particularly strong, including intelligent physical examination machines, smart watches, and so on; older people do not know how to use them… placing orders on mobile phones, many older people cannot do that either. (Participant 2, center staff)*

#### Communication barriers

Service providers think that most older adults and their family members are very friendly in communication, but conflicts also occur sometimes, blocking the services supply process. Domestic workers and nursing workers experience these negative experiences many times during their home visits:

*Some older people and their children think it (Home Care Service Voucher) is a free service that they can use at no expense. They make me do everything exceeding the amount of the voucher… I had to explain repeatedly, and some didn’t listen to me, thinking I was cheating… This kind of customer is too troublesome. (Participant 13, domestic worker)*

Some of the free services are targeted at a specific demographic. However, some families are not aware of the eligibility requirements of welfare beneficiaries, making judgment only by subjective comparison with the beneficiaries. In this way, many families think they should also receive free services. In order to obtain these services, they repeatedly communicate with the center manager and staffs through “complaint”, “comparison” and other ways. This kind of behavior also adds many additional communication problems for center managers and center staff:

*Some families come to us because they think they are poor, but others have free services, and they do not. It’s not our decision, and we have to explain it over and over again. (Participant 1, center staff)*

### Career dissatisfaction

In addition to the barriers mentioned above, staff members’ dissatisfaction with their careers inhibits quality service provision. They think that the salary is low and unstable, the task at hand is heavy, social acceptance of their occupation is poor, and there is no professional value, so they are not enthusiastic about the work (Table 2).

#### Low and unstable wages

Participants also raised concerns about wages and future job prospects, particularly for center managers and other center staff. Most of their average wages are about 2,000 yuan, which is not high. Due to the barriers caused by the COVID-19 pandemic, the instability of government financial support and the depression of the care market, HCSCs are facing a survival crisis. Therefore, pay cuts and layoffs are inevitable trends. These workers are in an uncertain work environment, which significantly affects their passion for work:

*From 2020, I started to receive half the basic salary. What’s worse, some of my colleagues have been laid off. There were six center staff members in our HCSC before, but now there are only two. Some HCSCs in our company are ready to close. I don’t know what’s going to happen… (Participant 3, center staff)*.

#### Heavy tasks

The ratio of service providers to customers was unbalanced, making it challenging to complete all work tasks. As one center staff member said:

*We have only two staff members and about 10 volunteers, but there are more than 2,000 older adults in our community. (Participant 2, center staff)*

They describe that it is hard to provide all older people with care services such as health check-ups, rehabilitation assessments, setting up Internet health records, buying medicines, and cleaning services with the limited staff:

*It’s laborious to provide door-to-door services, and we have to run around the city. We work from morning to night, cleaning windows, cleaning the cooker hood, etc. It’s very tiring. (Participant 28, domestic worker)*

As a result, many interviewees pointed out that:

*It’s impossible to complete all the service provision, and many are just going through the motions. (Participant 9, center staff)*

This formalistic approach is not conducive to IBHCS delivery.

#### Poor social acceptance of occupations

The dissatisfaction of domestic and nursing workers with the social acceptance of their occupations, which greatly affects their motivation at work, is a factor that they often mention during the interviews. Many managers described the situation; here is an example:

*It is very difficult to recruit nursing workers and domestic workers. Although we offer food and accommodation and a high salary of 6,000–10,000 yuan, there are still very few people applying. (Participant 10, manager)*

The results appear to be primarily due to occupational characteristics. Some domestic and nursing workers commented about this; here is an example:

*Others look down on this job and look down on me. I do very dirty and tiring work. I spend the whole day on the road and at customers’ houses. I cannot earn much money, but I am often made angry. If I can find another job, I don’t want to do this anymore. (Participant 28, domestic worker)*

#### Lack of professional value

The Internet-centric service delivery model ignores the value of professional staff, reducing their work enthusiasm. After the Internet was involved in home care services, the focus of service supply was placed on the application of new Internet technology. The advantage of Internet technology is that it realizes resource integration and more convenient demand transmission, but it is still inseparable from workers’ efforts. This tendency to focus exclusively on Internet technology creates an ethical dilemma in which the functions and values of service workers are ignored. Some interviewees expressed concern about the trend:

*I am not saying that the Internet is terrible. However, no matter how advanced Internet technology is, it also needs the support of offline platforms. With the current emphasis on technology, sometimes the efforts we put into our work are not rewarded, and I feel upset. (Participant 32, center staff)*

## Discussion

In this study, we explored the experience of service providers who provide IBHCS to older adults in urban China, and the challenges they face. We also provide further improvement strategies for policy and practice.

### Barriers to providing IBHCS for urban older adults

In China, the supply of IBHCS faces many challenges. From the perspective of service providers, this study found that service providers have multiple interactions with the government, the market, and older customers in service delivery, as well as encountering negative professional attitudes. Four barriers were identified: bureaucratic repression, profitability crisis in the market, non-cooperative clients, and career dissatisfaction. This perspective not only summarizes most of the barriers and reasons from the demand side through interaction with older adults. Moreover, we found more general obstacles that service providers are facing by placing them in the policy implementation and market environments under China’s unique authoritarian regime, which supplements the deeper analysis of the social structure constraints of why older adults’ needs cannot be met.

Firstly, China’s centralized bureaucracy is a significant obstacle, with unreasonable policy plans, harsh assessment, excessive paperwork, different preferences of government leaders, and the strict COVID-19 prevention and control measures making it difficult to provide IBHCS effectively. Several scholars have suggested that the government purchasing public services in China operates through strong bureaucratic management [36, 37, 66]. This approach will shift the focus of work from meeting older adults’ needs to meeting the government’s requirements, thus undermining service supply. Compared with the service users’ approach, the “bureaucratic repression” theme indicates that the service providers serve the government rather than older adults in practice [67]. Our study confirms the existing study results in general, but refines the barriers into different manifestations, enabling us to better understand them. Previous reports demonstrated that local governments set standardized implementation rules and quantifiable assessment indicators, and service providers gave priority to completing assessment tasks, resulting in service shortages [32, 35]. In other countries, such as Germany and Poland, caregivers have a disproportionate amount of time allocated to organizing documents, completing complex bureaucratic procedures, and providing services [68, 69]. In addition, Chen and Zheng found that service providers tend to change service items frequently to meet the preferences of purchasers and local officials, which makes it difficult to provide detailed, sustainable and in-depth services [70]. Besides, our study further sheds light on impediments to IBHCS due to the central tasks of combating COVID-19. The reduction or cessation of elderly care services due to the pandemic is an essential trend in all countries [71], especially in China, which has implemented strict prevention and control measures.

Secondly, we have noted that the profitability crisis in the elderly care market has also created a significant challenge to the supply of IBHCS, including high service costs, dampened effective demand, government intervention in setting prices, and parent companies’ excessively high sales targets. China’s elderly care industry is in its infancy and has not yet formed an intensive business model [72]. It is hard to keep a balance between supply and demand in an immature market, which thus results in restricted service supply [72]. The high costs lead to the high price of various services in IBHCS. Therefore, this leads to the financial distress commonly mentioned by users [73]. Under market competition, a large amount of research and development expense increases service cost [40, 74], which to a large extent dampens the enthusiasm of customers to consume. Older people’s low pensions and the traditional accumulation concept over consumption are deeply rooted [43], restricting their willingness to consume. We confirm Zhang et al.‘s view that elderly care services in China are a quasi-public service rather than a commercial service as in western countries [36]. However, interviewees noted that the price at which the government purchases services is significantly lower than the market price, not similar [36]. Therefore, most HCSCs can only basically survive or even lose money, which inhibits an improvement in the diversity of services. This study has further supplemented the experience of front-line staff. Parent companies’ excessively high sales targets have resulted in a large number of goal replacements, and the care services of older adults were crowded out.

In addition, client-related challenges also restrict the provision of IBHCS, which is embodied in the crisis of confidence, the dilemma of popularizing new technology, and communication barriers. This theme focuses on the interaction between service providers and service users, validating the numerous findings from the perspective of service users. As previously mentioned, privacy and security issues [41, 42], low adaptation to new things [39], and technology availability barriers [38] lead to a mismatch between the two. Chen proposed that in the process of public service supply, suppliers cannot match clients’ consumption attitudes and actual demands, so a large amount of service supply is rejected [75]. At present, elderly care companies face an insurmountable obstacle in a crisis of confidence caused by cyber fraud and security problems [76]. Although the literature in developed countries shows that older adults are willing to actively learn how to use new technologies to address the information gap [77], the service providers in this study experienced that older people’s rejection of new technologies and products is still strong. The poor interaction, communication, and cooperation between service providers and older adults further affect the efficiency of service supply [58, 78].

Lastly, the job dissatisfaction of front-line staff also affects their work enthusiasm. The occupational characteristics of low and unstable salaries, heavy tasks, poor social acceptance of occupations, and lack of professional value bring great challenges to service supply. Through the quantitative method, Zheng and Liu pointed out that job satisfaction has a significant positive impact on job performance [79]. Salary, workload, and professional identity affect the job satisfaction of front-line workers [80], and consequently affect work performance. A study from South Africa showed that caregivers face poor employment conditions, negative relationships, and role overload [81]. According to research by the Social Welfare Center of the Ministry of Civil Affairs, workers providing care services are characterized by high turnover rate, high labor intensity, low wage income, low social status, and low service level [82]. Our participants, especially young ones with excellent management and technical skills, also admitted their frustration that the Internet-centric service delivery model had erased the function and value of workers. This finding has not been mentioned often in previous studies.

### Policy and practical implications

In order to provide IBHCS better, we need to address the challenges service providers face. The main recommendations of this study are as follows:

Firstly, the governance environment of the IBHCS should be changed. We can consider developing more rational and dynamic rules and performance measurements, reducing paperwork and setting up longer project cycles. We should encourage the involvement of older adults in the future design of IBHCS, and government accountability in policy implementation should be strengthened, especially from the bottom up, to avoid different preferences of government leaders. Also, the advantages afforded by the Internet should be utilized to design a set of service supply methods to be applied during the COVID-19 and post-COVID-19 pandemic.

Secondly, fostering a mature elderly care market is the key. We should reduce service costs and expand revenue through more generous financial support, resource integration, technical assistance, and reasonable incentives. Specifically, the government has to encourage high-tech Internet enterprises from all aspects to develop an Internet-based elderly care service management system and application system that meet the actual needs of older adults. The government should support relevant high-tech enterprises to develop various intelligent devices suitable for elderly care services, for example, smart blood glucose meters, smart blood pressure meters, smartphones, smart bathing machines, and related mobile apps, to create a sufficient hardware foundation for older adults to enjoy high-quality and convenient care services. What’s more, we should innovate the way of elderly care services through diversified cooperation to achieve a win-win situation of convenient care service for older adults and enterprise profit.

Third, it is important to maintain good communication with older adults and determine their real needs. We suggest strengthening publicity and communication and establishing the correct cognition of IBHCS to reduce rejection behaviors. Meanwhile, the “consumer-led” concept should be established to develop intelligent service products suitable for usage by older people.

Finally, the findings suggest that the front-line staff’s working status and mindset must be changed. As an active intervention approach, social care can help address the problem [69]. Service delivery will be facilitated by ensuring that staff quality, competence, and professionalism are enhanced by providing better remuneration, appropriate training, and value recognition in various ways. In addition, we can recruit and cultivate a group of excellent professional volunteers and social welfare organizations into the service supply of IBHCS to reduce the work pressure on current staff.

### Strengths and limitations

The strength of this study is that it is the first comprehensive, systematic generalization of the barriers faced by Chinese service providers in delivering IBHCS. We collected a large number of materials from the daily life experience of service providers in 14 HCSCs in Changchun, China, and extracted the research findings through standard interview procedures. These findings provide unique evidence for the improvement of IBHCS supply.

There are three limitations to this study. First, the participants are all from Changchun, and the adaptability of the findings of this paper to other regions needs further investigation. Second, the interviewees chose the workplace office as interview site, which may have limited free expression to some extent, even if we ensured that no third parties were present during the interviews. Third, we only included the views of service providers. In fact, different politicians and policymakers also have many battles in the policymaking process, which would hinder the adequate supply of services. Further exploration is needed in future.

## Conclusion

Using qualitative research methods, in this study we investigated the barriers faced by service providers in providing IBHCS for urban older adults in China, offering empirical evidence for relevant literature in the Chinese context. China’s strong bureaucratic system makes service providers tend to fulfill the requirements of the government or leaders, which hinders the progress of service supply. The immature elderly care service market has brought about a mismatch between supply and demand, and restricts service delivery. What’s more, in “supplier-oriented” mode, clients’ consumption attitudes and demands are challenging to meet, leading to client-related challenges. Besides, the job dissatisfaction of front-line workers also affects their job satisfaction and performance, further impeding the implementation of IBHCS. In order to better promote it, we should improve the institutional environment and market environment, strengthen publicity and communication, pay attention to customer needs, and adjust the working conditions of front-line workers. Our findings provide helpful knowledge for service providers as well as policymakers that will facilitate improvements in IBHCS.

## Electronic supplementary material

Below is the link to the electronic supplementary material.

Supplementary Material 1

Supplementary Material 2

Supplementary Material 3

## Data Availability

The datasets generated and/or analysed during the current study are not publicly available due to participant privacy but are available from the corresponding author on reasonable request.

## Abbreviations

IBHCS: Internet-Based Home Care Service
HCSC: Home Care Service Center
COREQ: The Consolidated Criteria for Reporting Qualitative study
