# Supplementary material for: Barriers to providing internet-based home care services for urban older adults in China: a qualitative study of the service providers
Source: BMC Geriatr. 2023 May 23;23:320. doi: 10.1186/s12877-023-04028-4 (PMC10203686; doi:10.1186/s12877-023-04028-4)
Supplement: Supplementary file 2 — Supplementary Material 2 [file 12877_2023_4028_MOESM2_ESM.doc]

**Supplementary file 2**

**The semi-structured interview guide**

1. What specific Internet-Based Home Care Services (IBHCS) do you currently provide?

2. Which of these services do you think are up to scratch and which are lacking in some way?

3. How do you provide IBHCS?

4. Can you tell me about the barriers you have encountered in providing services?

5. How do you interact with government departments (Civil Affairs Bureau / Sub-District Offices / Community Neighborhood Committees)? What are the barriers? And why?

6. How do you interact with the care service market? What are the barriers? And why?

7. How do you interact with older adults? What are the barriers? And why?

8. How do you interact with older adults' family members? What are the barriers? And why?

9. What do you think of your job? What are the barriers? And why?

10. How do you solve these difficulties in your work?

11. How do you want to overcome these barriers in future?

12. Are there any omissions or additions to the topics we are talking about today that you would like to point out?
