# Supplementary material for: Barriers to providing internet-based home care services for urban older adults in China: a qualitative study of the service providers
Source: BMC Geriatr. 2023 May 23;23:320. doi: 10.1186/s12877-023-04028-4 (PMC10203686; doi:10.1186/s12877-023-04028-4)
Supplement: Supplementary file 3 — Supplementary Material 3 [file 12877_2023_4028_MOESM3_ESM.doc]

**Supplementary file 3: The main points of view for each respondent**

| **Case number** | **Job title** | **Theme** | **Sub-theme** | **Quotes** |
| --- | --- | --- | --- | --- |
| Participant 1 | Center staff | Bureaucratic repression | Unreasonable policy plans | Sometimes the policy design of the government is similar to that of the developed countries, such as making a lot of popular equipment. But they don't quite fit the reality. Especially in Northeast China, the economy is relatively backward... without this consumption capacity. |
|  |  |  | Harsh assessment | We must perform our contractual obligations strictly following the government's requirements. Because in this way, we can continue to get the project (government purchase service) with more and more trust from the government. |
|  |  |  | Excessive paperwork | Now the main task is to fill in various information and write plans. Because providing a service requires a highly complex process, such as writing plans, process records, and result records and filling them in the system... It is very troublesome. |
|  |  |  | Obstacles caused by COVID-19 control | During the epidemic, we suspended external services for older adults' safety. |
|  |  | Profitability crisis in the market | High service cost | I think the cost is too high. These types of high-tech equipment are pretty expensive. |
|  |  |  | Dampened effective demand | How to say, the demand for free services is quite great. As long as you have to spend money, there is no need. That is why the elderly care market is very difficult to develop. |
|  |  | Client-related challenges | Crisis of confidence | In the beginning, older adults distrusted us, thinking we were selling products, and not even talking to us. Then it was a little better because the community neighborhood committee helped us publicize it. |
|  |  |  | Dilemma of popularizing new technology | Another point is the digital divide that we often talk about now. Older adults can't use these new things and don't like to use them. |
|  |  |  | Communication barriers | Some families come to us because they think they are poor, but others have free services, and they do not. It's not our decision, and we have to explain it over and over again. |
|  |  | Career dissatisfaction | Low and unstable wages | The current salary has not reached the previous level. In general, the number of employees is either reduced or the remuneration is cut. Several HCSCs have been closed. |
| Participant 2 | Center staff | Bureaucratic repression | Harsh assessment | The parent company sent us a lot of government documents, such as government purchase contracts and assessment requirements. There are many items which must be strictly implemented. |
|  |  |  | Excessive paperwork | Now I feel that the administration of HCSC is relatively serious. Like the administrative department, various written files are very complex. I feel that we are the same as the staff in the Sub-District Office and Community Neighborhood Committee, except for the different responsibilities at work. |
|  |  |  | Obstacles caused by COVID-19 control | At present, the COVID-19 epidemic is relatively serious, and a lot of services cannot be carried out because of the closure and control of the epidemic. Unless at very special circumstances, older people and their families dare not let us enter the house. |
|  |  | Profitability crisis in the market | Dampened effective demand | At present, the " home care service voucher " project is one to make money. The demand for other charging items is deficient.. |
|  |  |  | Parent companies' excessively high sales targets | Our purchasing group has some children of older adults under 60 years old, or has brought in other relatives. Such purchasing capacity is strong, and the monthly performance is good. |
|  |  | Client-related challenges | Crisis of confidence | There are too many cases of older people being cheated, so they are very wary of strangers. Why do we always help the Community Neighborhood Committee? On the one hand, it is to serve the community residents together. On the other hand, it is to maintain good relations with the Community Neighborhood Committee and let them take the initiative to eliminate the vigilance of older adults. Older adults only trust government departments. |
|  |  |  | Dilemma of popularizing new technology | The willingness of older adults to accept Internet products is not particularly strong, including intelligent physical examination machines, smart watches, and so on; older people do not know how to use them... placing orders on mobile phones, many older people cannot do that either. |
|  |  |  | Communication barriers | Some older people are not very good at communication, which makes us feel headaches. But this is a minority. |
|  |  | Career dissatisfaction | Heavy tasks | We have only two staff members and about 10 volunteers, but more than 2,000 older adults are in our community. |
| Participant 3 | Center staff | Bureaucratic repression | Unreasonable policy plans | These (Internet) types of equipment are not used in the service but are used primarily to finish administrative work. They are invested in qualifying for government purchasing public services. However, most of them are not very useful, so they are just placed here and left unused. |
|  |  |  | Different preferences of government leaders | You have to listen to the (government’s) leaders and have a good relationship with them. What are the benefits? In that case, they will give it (the community with more services demand) to you because there are many older adults in this community. |
|  |  |  | Obstacles caused by COVID-19 control | Because of the currently closed management during the epidemic period, our canteen for older adults is also closed. |
|  |  | Profitability crisis in the market | High service cost | The biggest problem now should be the operation of elderly care companies, including HCSCs. The cost of the IBHCS program is high, but the consumption power of older adults is relatively poor. When prices are high, older people don't buy them at all. If the price is low, we lose money. |
|  |  |  | Dampened effective demand | The biggest problem now should be the operation of elderly care companies, including HCSCs. The cost of the IBHCS program is high, but the consumption power of older adults is relatively poor. When prices are high, older people don't buy them at all. If the price is low, we lose money. |
|  |  | Client-related challenges | Dilemma of popularizing new technology | At first, we made a smartwatch, but the older people were not willing to wear it. Later, we put a "button" on the body of old people, but the equipment was not very accurate. |
|  |  | Career dissatisfaction | Low and unstable wages | In 2020, I started to receive half the basic salary. What's worse, some of my colleagues have been laid off. There were six center staff members in our HCSC before, but now only two exist. Some HCSCs in our company are ready to close. I don't know what's going to happen... |
| Participant 4 | Center staff | Bureaucratic repression | Unreasonable policy plans | I majored in social work at my university, and have been engaged in elderly care services since graduation. I feel that the government has given us a lot of rules and regulations. Sometimes they are not particularly consistent with what we want to do or with the actual situation. I feel very tangled. |
|  |  |  | Different preferences of government leaders | We need to have a good relationship with the Community Neighborhood Committee; thus, we can communicate with older adults more smoothly. Older adults believe in the words of the Community Neighborhood Committee. In this way, it is beneficial to our work. |
|  |  |  | Obstacles caused by COVID-19 control | In the beginning, the "free clinic" activity was very popular. The community hospital also actively helped us prepare and publicize. We also received support from various government departments. However, it was delayed and stopped because of the epidemic. |
|  |  | Profitability crisis in the market | Dampened effective demand | Older people are in lower need. Maybe the pension is not high. This generation of older people is also used to saving, not willing to spend money. |
|  |  |  | Government intervention in setting price | The price is very low. Now there is a large demand for the "home care service voucher" project, which is profitable. |
|  |  | Client-related challenges | Dilemma of popularizing new technology | The original intention of IBHCS is good, and it is also the future development trend. But the adaptation problem of older adults needs to be addressed. For example, with a mobile phone, young people can learn very quickly. But you need to teach an older person for a long time. And the second day, he may forget it and come to learn it again. It may be repeated over and again. |
|  |  |  | Communication barriers | Some older adults are not talking about their actual needs. Some people are willing to "sadfishing" to get services. |
|  |  | Career dissatisfaction | Low and unstable wages | The salary is very low. I wouldn't do this job if I didn't want to give full play to my professional skills. Like our classmates, few of them are engaged in the field of social work in the end. They all feel that they do not make money and have no future. |
|  |  |  | Lack of professional value | I think our work is to serve others. We are all together because of the feeling of helping others. I hope society can recognize us more and see our professional value. |
| Participant 5 | Center staff | Bureaucratic repression | Harsh assessment | Assessments are very rigorous. For example, sometimes we need photo taking, video taking, and uploading them as evidence of providing services. Sometimes the teacher doesn't want to be photographed. In order to get money, we must do these. |
|  |  |  | Obstacles caused by COVID-19 control | The impact of the COVID-19 epidemic on HCSC is very large, and many services have been stopped. I don't know how long this HCSC will last. |
|  |  | Profitability crisis in the market | High service cost | At first, we all provided smart wristbands and smart sensors, but now we don't, because it's a money-losing project... Developing a wristband requires too much money. A wristband costs more than 300 Yuan, and the older people think it is too expensive, so no one buys it. |
|  |  |  | Government intervention in setting price | Government purchase price is relatively low, in order to enable older adults to enjoy more services. Our profits are very low and we need to make profits by volume. |
|  |  | Client-related challenges | Dilemma of popularizing new technology | These high-tech products are not used by older adults. This kind of physical examination machine, if we help them operate, older adults are willing to join in the experience. Other products, such as smart bracelets and smart mattresses, are not used by older adults. They don’t know how to use them. |
|  |  |  | Communication barriers | Our important service objects are disabled, demented older adults. As you know, this kind of older adults are generally not in good health, so it is difficult to communicate with them. It is much harder than communicating with others. |
|  |  | Career dissatisfaction | Low and unstable wages | They can't pay our wages any more. The HCSC is ready to close and has started to count the materials. |
| Participant 6 | Center manager | Bureaucratic repression | Excessive paperwork | Now there are too many checks, and there is no time for service. Different departments come to us to fill in forms…the same contents will be filled in several times... Whether we do it or not, we must have all the materials needed to look good. |
|  |  | Profitability crisis in the market | High service cost | Smart devices are expensive, but there are no subsidies. Moreover, the labor cost is also very high. |
|  |  |  | Government intervention in setting price | IBHCS is really not profitable. The price of the services purchased by the government is too low. Our HCSC can't make a lot of profits at all. |
|  |  | Career dissatisfaction | Low and unstable wages | Low wages... no way, now HCSC is losing money. |
| Participant 7 | Domestic worker | Bureaucratic repression | Obstacles caused by COVID-19 control | The workload was seriously affected by the COVID-epidemic. Because now if someone is infected or in touch with the infected, we have to close the community. And even now we dare not let outsiders come in, for fear of infection. |
|  |  | Career dissatisfaction | Low and unstable wages | We have a basic wage. Other income mainly depends on the service volume. You earn more if you do more, and less if you do less. |
|  |  |  | Heavy tasks | Without education and certificate, we have to do physical work. No physical work is not tiring. You can't earn money unless you do tiring work. |
| Participant 8 | Center manager | Bureaucratic repression | Unreasonable policy plans | Now the service is not very targeted. For example, there are a lot of mental consolation needs in our community. However, the government requires us to meet these needs mainly by holding regular lectures, which is not very effective... We cannot change it… |
|  |  |  | Harsh assessment | The government has clear requirements for the time and frequency of services. For example, he requires us to visit the house for no less than 40 minutes each time. In his system, you are not qualified if you punch in less than one minute and you can't get money. |
|  |  |  | Excessive paperwork | Now there is less time for front-line services, we are just writing bidding documents, taking projects and completing assessment forms at all stages. |
|  |  | Profitability crisis in the market | High service cost | I heard the company leaders say that the IBHCS is now particularly valued by the government, and the government regards us as a pilot project. In order to do it well, we have invested a lot of manpower, material resources and financial resources. |
|  |  |  | Dampened effective demand | Chinese older adults can't afford to spend money on themselves, but if you say to spend money on their children, they can afford it. Many older adults buy rice, flour, oil for their children with government subsidies. |
|  |  |  | Parent companies' excessively high sales targets | Our HCSC loses money every month. The boss pays for it. Who can stand it? So now the company has set tasks for us and mobilized us to overfulfill it every day. Alas, the pressure is very high. |
|  |  | Career dissatisfaction | Heavy tasks | There were seven staff in our store before. Some are responsible for connecting the hotline, some are responsible for providing specific services to older adults, and some are responsible for the daily work in the center. There are only two staff now, and the task is much heavier. |
|  |  |  | Poor social acceptance of occupations | Now it is difficult for us to recruit workers. Why? It is because this occupation is not respected. Once people hear that you are serving older adults, they look down on you. People would rather be waiters than do this. |
| Participant 9 | Center staff | Bureaucratic repression | Unreasonable policy plans | This room is equipped with cleaning and nursing beds, massage machines, etc. This is necessary for participating in the project of government purchase service. But in fact, no one uses it. This room is now used as a warehouse. |
|  |  |  | Excessive paperwork | Look at these files on my desk. They are all kinds of inspection materials, such as fire fighting, epidemic prevention, water and electricity safety. Now the rainy season is coming and there are flood control and flood prevention works. Too many files… |
|  |  |  | Dampened effective demand | In the beginning, health-monitoring equipment for medical care services was free, and everyone was willing to install it. When they are told they need to pay 160 yuan for installation, many older people are not willing to do it. Some said it's not helpful, some worried about privacy... There are all sorts of reasons. Actually, they just don't want to spend money. |
|  |  | Client-related challenges | Crisis of confidence | In our status, it is difficult for the older adults to trust us, and they think we always try to earn their money. If you tell him some knowledge of health care, he thinks you just want to sell things. |
|  |  |  | Dilemma of popularizing new technology | After all, an old man is old. Some things (intelligent devices) they can’t accept. When the phone rings, they don't know which button to press, let alone mobile order reservation service. |
|  |  |  | Communication barriers | Communication with the older adults is not like with young people, not a sentence or two sentences. You have to be patient and say it repeatedly, even so they may not understand it. |
|  |  | Career dissatisfaction | Low and unstable wages | Many of us have sideline business. Because of the epidemic, we open the door today and close the door tomorrow. The income is so unstable. There is no way to do it. We all have families and have to survive. |
|  |  |  | Heavy tasks | It's impossible to complete all the service provision, and many are just going through the motions. |
| Participant 10 | Manager | Bureaucratic repression | Unreasonable policy plans | The IBHCS was supposed to be a comprehensive service security net based on Internet technology, but now it is more like a domestic service. This is related to the government orientation, which pays more attention to solving the problem of daily care for the disabled older adults. |
|  |  |  | Harsh assessment | There is a special assessment department, which is very strict. If you fail to finish tasks or are complained, that's over. The next year you can't even win the bid. |
|  |  |  | Different preferences of government leaders | To put it bluntly, our main income comes from the purchase of services by the government, so we have to cooperate with their work. Some leaders like to do all kinds of activities and send news propaganda, some like to engage in infrastructure construction. You have to be clear about all these before you can win the bid. |
|  |  |  | Obstacles caused by COVID-19 control | Many people left their jobs during the COVID-19 pandemic. Once they leave, we need new employees to replace them and need to train the new group... It's too difficult. Several HCSCs have closed down. |
|  |  | Profitability crisis in the market | High service cost | The labor cost is very high. As I said, the salary of nursing workers and domestic workers is between 6000-10000 yuan. |
|  |  |  | Government intervention in setting price | The prices of our major services are all set by the government. If you want to increase the prices, it is very difficult. Now the cost is quickly rising, we are very hard. |
|  |  |  | Dampened effective demand | Our company started as a high-end company, serving those older adults who used to be senior cadres, with high pension and high requirements for living standards. Their working units also provide some subsidies and welfare, and the demand for purchasing services is very large. But now, we expand our service groups and cooperate with the government to do some inclusive projects. It becomes very difficult. Most older adults have too little pension to afford to spend. Chinese people are used to saving money all their lives, so it is too hard to pry their wallets. |
|  |  | Career dissatisfaction | Poor social acceptance of occupations | It is very difficult to recruit nursing workers and domestic workers. Although we offer food and accommodation and a high salary of 6,000-10,000 yuan, there are still very few people applying. |
| Participant 11 | Center manager | Bureaucratic repression | Obstacles caused by COVID-19 control | Due to the COVID-19 pandemic, the policy stipulates that door-to-door services for the elderly will be suspended. Older adults don't have a health code, so all these community activities such as free clinics have been suspended... so much work cannot be carried out now, and customer stickiness goes undiagnosed. Gosh, it is too hard… |
|  |  | Profitability crisis in the market | Government intervention in setting price | When the government bids, HCSCs must offer care services according to the bidding price. Some prices are set by enterprises and more often by the government. The government has greater bargaining power. |
|  |  |  | High service cost | Without subsidies, the development of (smart) wristbands is too expensive… |
|  |  | Client-related challenges | Crisis of confidence | We have large liquidity (of staff). They often quit after a period of work, so we have to recruit more people. The old man is slow to accept and recognize a person. He feels that he has just become familiar with you, and you will be replaced next month. Then he needs to be familiar with the new person, so he will not trust you. He always feels that you may quit at any time and won’t be responsible for him. He will find the Community Neighborhood Committee for anything. |
|  |  |  | Dilemma of popularizing new technology | Now there are too many smart devices and apps. We use this one, the Community Neighborhood Committee uses that one, and the Sub-District Office uses another. It is difficult even to teach older adults to operate one app. How can they accept so many? |
|  |  |  | Communication barriers | Older adults are somewhat deaf, and he can't understand what you say. You have to shout loudly to him when you are anxious. Some older adults think you have a bad attitude, and he will not be happy, let alone listening to you. |
| Participant 12 | Nursing worker | Bureaucratic repression | Obstacles caused by COVID-19 control | Later, due to the COVID-19 epidemic, the policy suspended the door-to-door service for older adults, so I rarely provide them now. |
|  |  | Career dissatisfaction | Low and unstable wages | The salary is too low. The main reason is that the time on the road isn’t counted, but it takes a lot of time to go back and forth. If it is far away, we can only pick up 2-3 orders a day. |
|  |  |  | Heavy tasks | If the old people in the family are disabled, they are usually taken care of by their children at home. Only for those who are particularly serious, they will pay for us to come home for care. We usually come half a month or a month, and they save all the work for us to do once. We are very tired every time. |
|  |  |  | Poor social acceptance of occupations | I am ashamed to tell others that I am a nursing worker. I am afraid that people will dislike me for being dirty. |
| Participant 13 | Domestic worker | Client-related challenges | Communication barriers | Some older people and their children think it (Home Care Service Voucher) is a free service that they can use at no expense. They make me do everything exceeding the amount of the voucher... I had to explain repeatedly, and some didn't listen to me, thinking I was cheating... This kind of customer is too troublesome. |
|  |  |  | Crisis of confidence | Now there are so many negative news on the Internet that their (older adults’) children don't trust us. We always feel being stared at when we work in their homes. |
|  |  | Career dissatisfaction | Low and unstable wages | I am an hourly worker, and my income is too unstable. If I have work, I can get money. If I have no work, I will have no income. I feel insecure every day. |
| Participant 14 | Center manager | Bureaucratic repression | Unreasonable policy plans | According to the plan, it will be started in December (2021)，and 4700 services are finished by March 2022. But the promotion is very difficult... it is impossible to achieve the goal. |
|  |  |  | Harsh assessment | The government requires us to take photos after completing the household service, but older adults see taking photos as a taboo. They often get unhappy because of taking photos, but there is no way for us because we’re required to do so. |
|  |  |  | Excessive paperwork | We need to keep a standing book for all the things in and out of the warehouse. We need to make a pile of materials for all the activities, and register various information for older adults. Alas, these things are very time-consuming, but we must still do them. So that if something happens in the future, we can also prove that we are following the rules. |
|  |  |  | Obstacles caused by COVID-19 control | The canteen service is particularly popular with older adults and is one of our main profitable programs. But now the epidemic control requires no in-room eating, we can only do food delivery, so the ordering is greatly reduced. |
|  |  | Profitability crisis in the market | High service cost | It's too expensive to hire professionals (domestic workers and nursing workers) now. We can only recruit some retired people, so we can save a lot of money without paying the “wuxianyijin” (it means the social insurances and housing fund). |
|  |  |  | Dampened effective demand | There are a lot of older adults who need care services. It seems there is much demand. However, they do not meet the eligibility criteria for a free Home Care Service Voucher, and the elderly have low pensions, so few of them are willing to buy it. |
|  |  |  | Parent companies' excessively high sales targets | The company has set a sales target for us, ranking every month, the ones lagging behind will get a deduction of money. However, the economic base of each district and the elderly group are different. How can we compete with others. |
|  |  | Client-related challenges | Crisis of confidence | The biggest difficulty is to win the trust of older adults. They always think that we are good to them in order to sell things and earn their money. Usually, our relationship is very good. Once money is involved, it is not easy to deal with it. They still cannot fully trust us. |
| Participant 15 | Manager | Bureaucratic repression | Harsh assessment | The meal services, we have stopped now. Because the assessment requires safe, hot meals, many older adults live far away, and it's challenging to accomplish... It’s too risky, and we could even lose the qualification (government procurement of services). |
|  |  |  | Different preferences of government leaders | After the old (Community Neighborhood Committee) secretary retired, he directly took the center away and it no longer belonged to the Community Neighborhood Committee. When the new secretary comes up, he will naturally consider whether the older one is practical and necessary. |
|  |  |  | Obstacles caused by COVID-19 control | During the COVID-19 epidemic, face-to-face contact and gathering were not allowed, and many services could not be carried out. We not only lost a lot of income, but also many excellent employees chose to leave or even change jobs because of income problems. |
|  |  | Profitability crisis in the market | High service cost | Now the cost is too high, and you have to spend money everywhere. |
|  |  |  | Dampened effective demand | Influenced by traditional ideas, most older adults still feel that it is a luxury to spend money on services. They would rather be suffering than to spend money. |
|  |  | Client-related challenges | Dilemma of popularizing new technology | We spent a lot of money designing app easy for older adults, and held many public lectures to help them adapt to the new technology, but the effect is not very good. They forget how to use it if they don't use it often. |
|  |  | Career dissatisfaction | Poor social acceptance of occupations | It is difficult to recruit nursing workers now. The labor cost is high. Although we can afford food and shelter, there are still few applicants. |
| Participant 16 | Center staff | Profitability crisis in the market | Dampened effective demand | Many old people don't like us touching their things and entering their houses. So even if he has some needs to be taken care of, he doesn't want strangers to come in. |
|  |  | Client-related challenges | Communication barriers | Now it is also the pilot stage, the policy is often adjusted. Some services are free before, but now charged. To explain it to older adults is particularly troublesome, they do not understand, thinking we are cheating them. |
|  |  | Career dissatisfaction | Low and unstable wages | We have a very low income, and there is no overtime pay for overtime work. If older adults have something, they will look for you regardless of whether it is working hours. They often call me at night. My family wants me to change my job. |
|  |  |  | Heavy tasks | It used to be five people who were responsible for this HCSC, but now there are only two of us. We can't cope with it at all. We have to prepare all kinds of materials and deal with all kinds of inspections every day. How can we afford to provide services again? We all have families and our own lives. |
| Participant 17 | Nursing worker | Bureaucratic repression | Obstacles caused by COVID-19 control | Many older adults have not been vaccinated. Even when the COVID-19 epidemic control is relatively loose, it is difficult for us to receive the order. They are worried that we will infect them. |
|  |  | Client-related challenges | Crisis of confidence | In fact, there is some risk to help older adults bathe. Many of their children are worried that we are not professional enough. They think that taking a bath may cause cold and it will aggravate the illness, so they will allow us to do some simple wiping. |
|  |  | Career dissatisfaction | Heavy tasks | The partially disabled older adults are better, but the completely disabled are particularly difficult to care for, which requires us to have strength as well as gentle movements. I often sweat all over doing that. |
|  |  |  | Poor social acceptance of occupations | Only older people are willing to do this job. Young people feel it dirty, being despised by others and so they are unwilling to do it at all. |
| Participant 18 | Center staff | Bureaucratic repression | Excessive paperwork | I have to spend three or four hours a day to complete all kinds of records, information forms, evaluation forms, signing forms and so on, which is very cumbersome. |
|  |  |  | Obstacles caused by COVID-19 control | During the COVID-19 epidemic, a lot of work cannot be carried out, and it is difficult to build customer stickiness. |
|  |  | Client-related challenges | Dampened effective demand | We have daytime hosting services here, but it's embarrassing that no one needs them. The disabled older adults cannot be picked up and sent off every day, and older adults without disability are unwilling to come, preferring to stay at home in a familiar environment. |
|  |  | Career dissatisfaction | Low and unstable wages | The salary is very low, about 2000 yuan. If it weren't for the poor employment environment, I would probably resign. |
| Participant 19 | Center staff | Bureaucratic repression | Harsh assessment | Very strict... There will be monitoring of the service provider's personnel allocation, qualifications and capabilities, service quality and service risk. Later, it is required to build a platform and auxiliary facilities for older adults. |
|  |  |  | Obstacles caused by COVID-19 control | We are not allowed to gather during the epidemic. Many of our promotional activities and health lectures cannot be carried out. It is difficult to complete the sales volume. |
|  |  | Profitability crisis in the market | High service cost | We spend a lot of money on these facilities. You see, these beds, kitchens, showers, and massage equipment are all money, but they are rarely used. The cost is hard to recover. |
|  |  |  | Parent companies' excessively high sales targets | Every month, the parent company supervises us to complete the tasks, but our publicity funds are limited, so it is difficult to expand customers, and the pressure is very great. |
|  |  | Client-related challenges | Dilemma of popularizing new technology | Older adults may have an instinctive resistance to new technologies. They don't like to learn to use new technologies. |
|  |  |  | Crisis of confidence | There are some services, for which older adults need to provide home address, ID card and other information. They are very sensitive, and feel that those who want this information are cheaters. |
| Participant 20 | Manager | Bureaucratic repression | Unreasonable policy plans | At the time of bidding, we were required to have beds and bath equipment. We spent money to buy nursing beds. But there are not older adults willing to live here. The equipment only stays idle. |
|  |  |  | Harsh assessment | The bidding qualification is very strict, and there are very detailed requirements for the operating years, investment capital, staff, housing area and safety facilities. We have been preparing for a long time to meet the standards. |
|  |  |  | Different preferences of government leaders | They (the community neighborhood committee) change leaders all the time, almost every two years. A new broom sweeps clean. The new leadership will naturally consider whether the existing project is practical, or good, whether it is necessary... We have to listen to their ideas to stop some projects and develop some new ones. |
|  |  | Profitability crisis in the market | High service cost | We have to consider the market reputation, so we invest a lot. |
|  |  |  | Government intervention in setting price | Several major services are priced by the government and are basically not profitable. We can only rely on these services to accumulate reputation, develop customers, and then increase revenue through other charged services. |
|  |  | Client-related challenges | Crisis of confidence | A lot of work needs to be done depending on the Community Neighborhood Committee, because older adults do not trust us. |
|  |  |  | Dilemma of popularizing new technology | We have developed a lot of software and electronic monitoring equipment to prevent older adults from accidents. But they always feel that someone is monitoring. They feel uncomfortable and refuse to use it. |
| Participant 21 | Domestic worker | Bureaucratic repression | Obstacles caused by COVID-19 control | During the epidemic, either I was isolated at home or the customer was isolated at home, so it was difficult to receive the order. |
|  |  | Client-related challenges | Crisis of confidence | Building trust between strangers is the most difficult. What's more, I have to work at older adults’ home. When they are alone at home, they will not let us provide door-to-door service. So, a large number of orders are accumulated at the weekend. |
|  |  | Career dissatisfaction | Heavy tasks | Older adults think we should obey their orders since they spend money. They often ask us for additional requirements, such as buying milk on the way to their houses, helping to throw garbage out and tidy up the yard when leaving. |
|  |  |  | Poor social acceptance of occupations | Everyone thinks that domestic workers are low in technical content, being ordered by others, and with low status. When I talk about my occupation with others, I will feel embarrassed. |
| Participant 22 | Center manager | Bureaucratic repression | Unreasonable policy plans | The government's policy requirements are too rigid, such as the unified provision of care services. This makes us inflexible in the supply of services, making it difficult to meet the diverse needs of older adults in the community. |
|  |  |  | Excessive paperwork | In my daily work, I need to deal with a lot of paperwork, which makes me feel disgusted. But there is no way. These materials are used for (government) inspection and assessment. |
|  |  |  | Obstacles caused by COVID-19 control | The COVID-19 pandemic has affected our business just too much. Most of the time, we cannot go to the home to provide services for older adults. This makes me very worried that over time, we will become unfamiliar in our feelings, and we may lose our old customers. |
|  |  | Profitability crisis in the market | High service cost | At the beginning, in order to show the characteristics of "smart", we purchased a large number of smart wearable devices for older adults. Later, we found that the elderly would not use them or thought it was too expensive to buy. Most of these devices are now stacked in warehouses. |
|  |  |  | Dampened effective demand | Older adults are reluctant to spend money, and there are few government-supported free services. Effective demand has not been activated very well. |
|  |  |  | Government intervention in setting price | The government's pricing is too low, and the operation of HCSC is very difficult. So we cut down some service items and mainly provide services with higher profits. |
|  |  | Client-related challenges | Crisis of confidence | Community residents don't know much about IBHCS. We are often considered to be cheaters and it is difficult to carry out business. |
|  |  | Career dissatisfaction | Low and unstable wages | At present, our main source of income is government purchase services, which is single. This also leads to our inability to make competitive salary incentive policies. Everyone's salary is relatively fixed and not high. If we can't get the government project, it will be difficult for us to have money. |
| Participant 23 | Center staff | Bureaucratic repression | Harsh assessment | Now our work, in short, is to complete government tasks, doing some fake tasks, and reaching the standards with pictures and video evidence. |
|  |  |  | Excessive paperwork | I often need to assist the center manager to sort out a lot of paperwork to cope with the inspection, which takes up almost 1/3 of my daily work time. |
|  |  | Profitability crisis in the market | High service cost | Our HCSC bought a lot of smart devices, spending a lot of money, but now many of them are left idle. |
|  |  |  | Dampened effective demand | Demand for many services is still very low, for example, assisted bathing. At the beginning, the purchasing agent service was not good, but the COVID-19 epidemic raised the demand for it. |
|  |  | Client-related challenges | Dilemma of popularizing new technology | Older adults are not very interested in smart watches and smart phones, because most of them can't use them. We teach them how to use it, but they think it is troublesome and can't learn it. |
|  |  |  | Communication barriers | In service, I was mostly asked "why can other people get free service and I don't." I often explain to them that the government policies have corresponding provisions, but many people don't understand it and sometimes we both get upset. |
|  |  | Career dissatisfaction | Low and unstable wages | The salary is a little low, just good enough to support myself alone. |
|  |  |  | Heavy tasks | I am responsible for hundreds of older adults’ service work alone, and I will hurry to where I am needed. I often need to work overtime. I am tired every day. |
| Participant 24 | Center manager | Bureaucratic repression | Unreasonable policy plans | We provide services by government purchasing. We should comply with the contract. It's just that some requirements are too detailed and difficult to operate. |
|  |  |  | Excessive paperwork | At present, the government's routine inspection is basically only looking at documents, so we usually need to mobilize all staff to sort out all documents. This one is just too time consuming. |
|  |  |  | Obstacles caused by COVID-19 control | During the COVID-19 epidemic, the Community Neighborhood Committee asked us to suspend all service operations. The impact on HCSC is almost fatal. |
|  |  | Profitability crisis in the market | High service cost | We also have those smart mattresses, smart sensors, smart wristband...and all. However, the use in front-line care is not ideal. Because when these things are added up, the consumption cost of older adults will immediately increase. The pension of older adults is about 2-3 thousand yuan, they can’t bear it. |
|  |  |  | Government intervention in setting price | The government price is too low for us to make money. For those with relatively low demand, with only 10-20 orders a year, we cannot reduce labor costs. |
|  |  | Client-related challenges | Crisis of confidence | Many older adults and their families do not trust us and are afraid that we are selling something. The older adults are also afraid of being cheated. Two years ago, a grandpa in this community was cheated thousands of yuan to buy health care products. There are too many bad people now. |
|  |  |  | Dilemma of popularizing new technology | I think the most important thing is that older adults do not know how to use these intelligent things. For example, they can't use mobile phones. They always come here to ask me how to use it. Some older people accidentally click a key and don't know how to switch it back. |
|  |  | Career dissatisfaction | Low and unstable wages | Poor pay is a common phenomenon in our industry. With the epidemic we are making even less money. |
|  |  |  | Heavy tasks | Very busy. Like other places, one HCSC is matched with three or four people. There are only two of us here. I take care of the supermarket. Ms. Li is responsible for other work in the center. And we are often called to help the Community Neighborhood Committee. |
| Participant 25 | Center staff | Bureaucratic repression | Unreasonable policy plans | These devices are all required to provide. Some are useful, such as this physical examination machine, at the beginning, a lot of older people came for a test. But nursing bed is not very useful. |
|  |  |  | Different preferences of government leaders | You must listen to the leaders of the Community Neighborhood Committee. It’s their place, so they have the final say. |
|  |  |  | Obstacles caused by COVID-19 control | Free medical treatment, community activities and other projects were all suspended in the first year of the COVID-19 epidemic. The epidemic situation is a little better now, but under the normalization of the epidemic situation, we would do it only several times in the summer. |
|  |  | Profitability crisis in the market | Parent companies' excessively high sales targets | The company sets targets according to each HCSC situation. The goals are high and often are not achieved...If you achieve the goal, the reward will be more. |
|  |  | Client-related challenges | Dilemma of popularizing new technology | It is difficult for older adults to learn such high-tech things. For example, some older people can't see clearly and have poor memory. They can’t even learn what we think easy. This is far too common at our HCSC. |
|  |  | Career dissatisfaction | Low and unstable wages | I am worried that the HCSC may closed. Several have been closed. During the current epidemic, no new job can be found. |
| Participant 26 | Center manager | Bureaucratic repression | Excessive paperwork | Our HCSC requires us to complete the travel forms, activity records, image materials and other data of the day every day. This is quite tedious. |
|  |  |  | Obstacles caused by COVID-19 control | The epidemic has almost put us out of work. All offline services were asked to stop during that time. |
|  |  | Profitability crisis in the market | High service cost | This (smart) system is expensive, costing tens of millions yuan. |
|  |  |  | Dampened effective demand | Now taking care of older adults is not like raising children for whom they are willing to spend any money. These people, comparatively, don’t like to spend more money on older adults. |
|  |  | Client-related challenges | Crisis of confidence | We are involved as a third-party organization. People don't recognize us, thinking we are liars. When we ask to use older adults' mobile phones to register and fill in their basic information, they refuse to comply.  Media opinion has a strong impact. There is much negative news about abuse of the elderly, or related subjects. In fact, such abuse is really rare. But the Chinese people love to pay attention to negative news involving accidents or something terrible. This makes some older people and their children distrust us greatly. |
|  |  |  | Dilemma of popularizing new technology | These older people can't even use their mobile phones. You ask them to install APP, or do this and that. They can't get it. |
|  |  | Career dissatisfaction | Low and unstable wages | Our wages are all about the same, a little more than 2000 yuan. The place is close to home, and you can take care of the family. You can't do this if you want to support your family. |
| Participant 27 | Center manager | Bureaucratic repression | Harsh assessment | The (government) requirements are too many. It depends on more than 30 indicators, such as equipment matching, risk control, service satisfaction, etc. If you fail to meet the requirements, you will be punished. Our leaders have stressed these indicators all day long for fear of problems. |
|  |  | Profitability crisis in the market | High service cost | Our smart devices are invested for government’s projects, not with government subsidies. So the cost is very high… |
|  |  | Client-related challenges | Crisis of confidence | We asked the older people to register the information. At first, everyone was reluctant, afraid that we would cheat them of money, so they don’t trust us. |
|  |  |  | Dilemma of popularizing new technology | The promotion of smart devices is the most troublesome thing for me. For example, older adults feel smart watches are inconvenient to carry and they cannot use them. |
| Participant 28 | Domestic worker | Career dissatisfaction | Heavy tasks | It's laborious to provide door-to-door services, and we have to run around the city. We work from morning to night, cleaning windows, cleaning the cooker hood, etc. It's very tiring. |
|  |  |  | Poor social acceptance of occupations | Others look down on this job and look down on me. I do very dirty and tiring work. I spend the whole day on the road and at customers' houses. I cannot earn much money, but I am often made angry. If I can find another job, I don't want to do this anymore. |
|  |  |  | Lack of professional value | I do very dirty and tiring work…If I can find another job, I don't want to do this anymore. |
| Participant 29 | Nursing worker | Client-related challenges | Crisis of confidence | When I came to the house to take care of older adults, I was often stared at by the family members with strange eyes, which made me feel very uncomfortable. |
|  |  |  | Communication barriers | Sometimes it’s very far away from the unit to the customer's home, so I cannot arrive at the customer's home at the specified time. Some older people ask you to come to take care of them within the specified time, otherwise they will complain. It is difficult to communicate with them. |
|  |  | Career dissatisfaction | Heavy tasks | Serving people is a hard job. Some old people who can't move, you have to turn them over. |
|  |  |  | Poor social acceptance of occupations | Compared with doctors and nurses, our nursing workers have no status. To put it plainly, we should do some work like helping older adults change their diapers and cleaning up their wounds. |
| Participant 30 | Center manager | Bureaucratic repression | Unreasonable policy plans | The IBHCS service content is single, and older adults have a poor experience. |
|  |  |  | Excessive paperwork | There are too many materials to sort out and submit. It's very troublesome. Sometimes we can't afford to provide services for older adults. |
|  |  |  | Obstacles caused by COVID-19 control | During the COVID-19 epidemic, government departments and the Community Neighborhood Committee required us not to enter the house to provide services for older adults. However, older adults with diseases need us to provide services at home, which makes it very difficult for us and it has a great impact on our work. |
|  |  | Profitability crisis in the market | Government intervention in setting price | The government has the document requirements about the charges for service at home. The HCSC cannot set the price at will, otherwise it will be punished (by the government). |
|  |  | Client-related challenges | Crisis of confidence | At present, the advertisements of commercial elderly care services are overwhelming. Many of them have false propaganda, and many older adults do not believe it. This has also led them to believe that the IBHCS is also unreliable. |
|  |  |  | Communication barriers | You have to be patient enough to communicate with older adults. These people always think they already understand when they are just half listening or don't understand at all. |
|  |  | Career dissatisfaction | Low and unstable wages | Our salary is generally more than 2000 to more than 3000 yuan a month, which is relatively low. |
| Participant 31 | Nursing worker | Client-related challenges | Crisis of confidence | Family members are still a lot wary of us. For example, they are worried that we will abuse older adults as reported by the news media. |
|  |  |  | Communication barriers | I'm from another place. Some local elderly people only speak local dialect, so I can't understand it. It's very inconvenient at this time. |
|  |  | Career dissatisfaction | Poor social acceptance of occupations | We mainly help the elderly patients wash their faces, wash their feet, feed, wipe their bodies, and take care of their urine and feces. We do some hard and dirty work, and young people are unwilling to do our work. |
| Participant 32 | Center staff | Bureaucratic repression | Harsh assessment | Some indicators are easy to complete, while others are difficult. For example, when we enter a house, we have to take photos and videos to prove that we have entered the house. Many families don't like it, so they tell us so. We have no way for it. We can't finish the task without taking pictures. |
|  |  |  | Excessive paperwork | Our current work is all very standardized. You need to fill in the service form when you enter the house. You need to fill in several forms when you serve 3-4 houses a day. These data of the day need to be filed and sorted in time when they are returned to the HCSC, which is very cumbersome. This is what they look at in the assessment, so we must do it well. |
|  |  |  | Different preferences of government leaders | Before doing some service, we ask the Sub-District Office and Community Neighborhood Committee what requirements they have and what ideas they have. They also have some related tasks. Sometimes we carry out a project and help them finish some work at our convenience. It's a win-win situation. |
|  |  |  | Obstacles caused by COVID-19 control | Now it is mainly the impact of the COVID-19 epidemic, and many services cannot be developed. Leaders also have headaches for this. |
|  |  | Profitability crisis in the market | Dampened effective demand | The consumption mainly comes from "home care service voucher", which some older people have 400 yuan monthly. |
|  |  |  | Parent companies' excessively high sales targets | At first, the purchasing agent service was aimed at older adults, but they do not have strong purchasing power like young people do. We were finding it hard to finish the task every month. So, we opened it up to young people for them to buy. Since then, the goal is easily accomplished. |
|  |  | Client-related challenges | Crisis of confidence | When we introduce these products, many older adults and their children always refuse us, afraid of being cheated again. |
|  |  |  | Dilemma of popularizing new technology | Older people are not particularly willing to accept smart products. For example, installing an Internet TV will have better function. But they still like cable TV, it's convenient to turn on two buttons, and that’s done. |
|  |  | Career dissatisfaction | Low and unstable wages | My salary is 2500 a month, which is too low. My parents advised me not to do it anymore. |
|  |  |  | Lack of professional value | I am not saying that the Internet is terrible. However, no matter how advanced Internet technology is, it also needs the support of offline platforms. With the current emphasis on technology, sometimes the efforts we put into our work are not rewarded, and I feel upset. |
| Participant 33 | Domestic worker | Client-related challenges | Crisis of confidence | When entering the house for service, some older people often remind me not to break the vase when cleaning, and clean up all the corners. They have doubts about our service quality. |
|  |  |  | Communication barriers | When doing the home service, we should pay special attention to communicating with customers, otherwise it is easy to be complained. |
|  |  | Career dissatisfaction | Heavy tasks | It usually takes 1-2 hours to clean a room at home. Sometimes I can take several orders a day. It's very tiring to do it all day. |
|  |  |  | Poor social acceptance of occupations | We do dirty and hard work. This occupation is not decent. |
| Participant 34 | Nursing worker | Client-related challenges | Crisis of confidence | Now many news reports about older adults beaten by the nursing worker. So that when I enter the house for service, the family members usually watch me when I do things, and they are worried that I will cause harm to older adults. I can understand that, but it makes me very uncomfortable. |
|  |  | Client-related challenges | Communication barriers | Some older people who have been ill for many years have strange personalities. Your careless words may touch their fragile nerves, so you have to be careful. |
|  |  | Career dissatisfaction | Poor social acceptance of occupations | Workers in my job are about the same age like me. No young people want to do it. I feel inferior when I work. |
